# Comparison digestibility and protozoa population of Khuzestan water buffalo and Holstein cow

**Published:** 2014

**Authors:** Safora Jabari, Moosa Eslami, Morteza Chaji, Tahereh Mohammadabadi, Mohammad Bojarpour

**Affiliations:** *Department of Animal Science, Faculty of Animal and Food Science, Khuzestan Ramin Agriculture and Natural Resources University, Mollasani, Ahvaz, Iran.*

**Keywords:** Ciliate protozoa, Gas production, Rumen fluid, Steam treated sugarcane pith

## Abstract

The major aim of this study was to compare the morphology and activity of rumen protozoa of Khuzestan water buffalo and Holstein cow using *in vitro* digestibility and gas production parameters of steam treated sugarcane pith. Rumen fluid obtained from two buffalo and cow steers fed the same diet, 30:70 concentrate: forage. To separate rumen protozoa, antibiotic solution and fungicides were added to rumen fluid. The results of present experiment indicated that the neutral detergent fiber (NDF; 7.8 *vs. *1.69%) and acid detergent fiber (ADF; 6.24 *vs.* 3.24%) digestibility of steam treated sugarcane pith by rumen protozoal population of Khuzestan buffalo was higher than those of cow (*p *< 0.05). Also, digestibility of dry matter, NDF and ADF by whole buffalo micro-organisms was more than those in cow (*p *< 0.05). The results indicated that the potential of gas production of sugarcane pith by rumen protozoa in water buffalo was more than that of cow (*p *< 0.05). Total rumen ciliate protozoa numbers in water buffalo were significantly higher than those of cow (3.68 × 10^5^ *vs.* 2.18 × 10^5^ mL^-1^ of rumen content) (*p *< 0.05). The number of *Diplodinium* in buffalo was more than that of cow (41.27 *vs.* 35.7% of total rumen protozoa, respectively). Percentage of *Entodinium*, *Epidinium*, *Ophryoscolex* and *Isotricha *in cow was more than those of buffalo. Therefore, in the same diet, protozoa and total rumen micro-organisms of Khuzestan water buffalo have higher digestion activity compared to Holstein cow.

## Introduction

Sugarcane pith, a by-product which remains after rind removal of sugarcane, is the most abundant by-product in southwest of Iran. Poor-quality forages apparently are digested more efficiently by bison than by cattle.^1^^,^^2^ Reportedly, buffalo digest fibrous feedstuff more efficiently than cattle, particularly with diets which have a high proportion of cellulose.^3^ The rumen of buffalo is well adapted to utilize the lignocellulose residues.^4^^,^^6^^,^^8^^,^^13^ It has been reported that when cattle and buffalo were kept under similar conditions, buffalo utilized feeds more efficiently (2 to 3%) than cattle.^5^ The mechanisms that are responsible for the putative differences in digestive capacity between bison and cattle have not been examined, but they may involve differences in ruminal microbial populations. Comparative studies on the digestive physiology of buffalo and cattle give varying results. Many authors attribute buffalo to bear a higher and greater contraction force in the rumen, a more intense activity of cellulolytic microflora.^6^^,^^8^^,^^10^ In contrast, other authors found a significantly lower *in situ *dry matter (DM) degradability and* in vitro *DM digestion or microbial pools in comparison with cattle.^7^^,^^8^ Comparative trials between the buffalo and Friesian cattle in Italy showed buffalo a better rumen degradability of fibrous fractions.^9^ Rumen microorganism of buffalo is further and more varied than the cattle.^10^ Grant *et al.* studied the influence of the rumen fluid source (Philippines cattle and buffalo) on *in vitro *true dry matter digestibility of forages, and reported that the digestibility was the same for the two inocula if the donor animals were consuming the same diet.^11^ Any variations between cattle and buffalo in proportions and numbers of ruminal bacteria, protozoa and fungi might contribute to the explanation of differences in digestive capability due to fermentation end products available for absorption and utilization by ruminants. The researches indicated that ruminal protozoal populations differed both quantitatively and qualitatively between buffalo and cattle. *In vitro* studies have suggested that 19.00 to 28.00% total cellulase activity can be attributed to protozoa.^12^ Also, Lee *et al.* reported that rumen protozoa cause 25.00 to 30.00% of total rumen microbial fiber digestion.^13^ Coleman showed that the ability of *Epidinium ecaudatum* to degrade microcrystalline cellulose was high.^14^ On the other hand, there are also evidence showing that the xylan and especially microcrystalline cellulose digesting and fermenting capacities of *Epidinium* isolated from the rumen are substantially lower than those of *Eudiplodinium maggii* and *Polyplastron multivesiculatum*.^15^ The objective of this study was to understand the possible differences between Khuzestan water buffalo and cattle by comparing *in vitro* ruminal degradation characteristics, digestibility, and protozoal populations in both species.

## Materials and Methods

**Preparation of inoculums. **Rumen fluid was taken before the morning feeding from two water buffalo and two Holstein cow (live weight around 450 kg) via stomach tube. Experimental animals were housed and maintained under similar conditions. All of them received the same diet, 30:70 concentrate (Corn grain, barley grain and wheat bran): forage (Sugarcane silage, corn silage, alfalfa hay and wheat straw), about 8 kg per day on DM base. Samples of rumen contents were collected separately into thermos flasks. Rumen fluid was strained through four layers of cheesecloth, and kept at 39 ˚C under CO_2_ condition, and used for *in vitro* experiment. To prepare rumen protozoa, antibiotic solution (streptomycin sulphate, penicillin G and chloramphenicol, 0.1 mg mL^-1 ^each; Sigma-Aldrich Co., Taufkirchen, Germany) and fungicides (Benomyl: 500 ppm mL^-1^ medium and metalaxyl: 10 mg mL^-1^ medium; Sigma-Aldrich Co., Taufkirchen, Germany) were added to rumen fluid.^16^

***In vitro*** **digestibility. **The *in vitro* digestibility measured by the *in vitro* procedure was modified from that reported by Tilley and Terry.^17^ Rumen fluid was collected from two water buffalo and Holstein cow, and isolated ruminal protozoa were mixed with McDougall buffer in a ratio 1:4. After gasifying with CO_2_, tubes were incubated at 39 ˚C.^18^ After 48 hr of fermentation, 6 mL of 20% HCl solution (Merck Co., Darmstadt, Germany) and 5 mL pepsin solution (Merck Co., Darmstadt, Germany) were added and the incubated for 48 hr simulating post-ruminal degradation. After incubation, the residual substrates of each tube were filtered and used to determine digestibility of dry matter (DM) and neutral detergent fiber (NDF). The obtained data were subjected to analysis as a completely randomized design using the General Linear Model (GLM). Duncan’s multiple range test was used to compare treatment means at *p* < 0.05.

***In vitro*** **gas production.** *In vitro* gas production technique was conducted according to the Menke and Steingass.^19^ Rumen fluid was obtained from two buffalos and two cows, and rumen protozoa were isolated. About 500 mg experimental sample (1.0 mm screen, sugarcane pith) were incubated with 30 mL buffered rumen fluid that was consisted of 10 mL of rumen liquor and 20 mL of buffering solution^19^ under continuous CO_2_ reflux in 100 mL calibrated glass syringes in a water bath maintained at 39 ˚C. Samples were incubated in triplicate together with three syringes containing only incubation medium (blank). Protozoa were isolated and flushed with CO_2_, then prepared rumen protozoa were added to the buffered solution (1:2 v/v), which was maintained in a water bath at 39 ˚C. The syringes and their contents were maintained at 39 ˚C in an incubator. *In vitro* gas production was determined at 2, 4, 8, 12, 24, 48, 72 and 96 hr after incubation of samples. 

**Protozoa enumeration. **The rumen fluid was collected from water buffalo and Holstein cow. The identification and counting of rumen ciliate protozoa were performed according to Ogimoto and Imai and Dehority under light microscope.^20^^,^^21^ Rumen fluid was obtained by stomach tube before the morning feeding, homogenized in a laboratory blender, filtered through four layers of cheese-cloth. Then, 10 mL samples were fixed in equal volume of 18.50% formaldehyde solution (Merck Co., Darmstadt, Germany) in glass jars properly labeled, sealed and kept in dark place at room temperature. The following genera were counted separately: *Entodinium*, *Epidinium*, *Isotricha*, *Dasytricha* and ciliates belonging to the subfamily *Diplodiniinae*, were counted together: *Metadinium*, *Eudiplodinium*, *Ostracodinium*, *Elytroplastron* and *Polyplastron*. After thorough homogenization of each sample, 1 mL was pipetted using special wide aperture pipette and placed in a test tube. Then two drops of 2.00% of brilliant green solution were added and kept overnight at room temperature. The subsequent dilutions were made with 30.00% glycerol solution (Merck Co., Darmstadt, Germany) according to concentration of cell number in the sample. The count in each sample was performed by Sedgewick-Rafter counting chamber (Pyser-SGI, Kent, UK) in an optical microscope at magnification 100×, with eyepiece grid containing 0.50 mm^2^. The samples were examined under a light microscope.^21^ Identification of ciliate protozoa was according to the description published by Ogimoto and Imai.^20^

**Statistical analysis. **After 96 hr of incubation, cumulative gas production data were fitted to the exponential equation: 

*Y=b (1−e* ^−ct^ *)*

where, *b* is volume of potential gas production from the fermentable fraction (mL), c is the gas production rate constant for *b* (mL per hr),* t* is the incubation time (hr) and *Y* is the gas volume produced at time *t*. Data of DM, NDF and ADF digestibility,* in vitro* gas production, and protozoa enumeration were analyzed as a completely randomized design using the GLM procedure of SAS (Version 8.2; SAS Institute, Carry, USA). Duncan’s multiple range test was used to compare treatment means at (*p* < 0.05).

## Results

Digestibility of DM, NDF and ADF during the incubation periods are given in Table 1. Results showed that there was a significant difference in *in vitro* NDF and ADF digestibility between rumen protozoa of buffalo and cow (*p *< 0.05). The results also indicated that NDF digestibility of steam treated sugarcane pith was 7.80 and 1.69% for protozoal population of buffalo and cow, respectively (*p *< 0.05). The DM and ADF digestibility (6.24 *vs.* 3.24%) of steam treated sugarcane pith by rumen protozoal of Khuzestan buffalo was higher than that of cow (*p *< 0.05). 

Gas production parameters during the incubation periods are given in Table 2. The results indicated that potential of gas production by water buffalo rumen protozoa was higher than that in cow (51.01 *vs*. 48.22 mL, respectively). However, gas production rate of buffalo rumen protozoa and cow was same (*p* > 0.05). Potential and rate of gas production by total rumen microbes in water buffalo were higher than that of cow (*p* < 0.05). In this study, a significant influence of rumen *inoculum* (water buffalo *vs*. cow) on fermentation and degradability of the examined samples was found. 

The concentration (cell number per mL of rumen content) and composition (as percentage of total) of ciliated protozoa in the rumen of buffalo and Holstein cow are shown in Table 3. Total rumen ciliate protozoal numbers were higher in buffalo than in cow (3.68 × 10^5^ *vs.* 2.18 × 10^5^), (*p* > 0.05). 

**Table 1:** *In vitro* digestibility of sugarcane pith by Khuzestan water buffalo and Holstein cow (n=8). Data are presented as mean ± SD.

	**Dry matter (%)**	**Neutral detergent fiber (%)**	**Acid detergent fiber (%)**
***Rumen protozoa***			
** Buffalo **	25.84 ± 0.42	7.80 ± 0.19 a	6.24 ± 1.94 a
** Cow **	25.22 ± 0.47	1.69 ± 0.67^ b^	3.24 ± 0.46 b
** SEM**	0.32	1.50	1.10
***Total rumen microorganisms***			
** Buffalo**	51.05 ± 1.46 a	49.17 ± 0.11 a	31.72 ± 2.97 a
** Cow**	38.80 ± 0.90^ b^	25.40 ± 0.41 b	20.25 ± 0.56 b
** SEM**	0.69	0.95	2.23

a,b Values within each column with different letters are significantly different (*p* < 0.05).

**Table 2:** Gas production parameters of sugarcane pith incubated by Khuzestan water buffalo and Holstein cow rumen liquid. Data are presented as mean ± SD.

	**Gas production volume (mL)**	**Gas production rate** ** (mL per hr)**	
***Rumen protozoa***			
Buffalo	51.01 ± 1.78	0.03 ± 0.03	
Cow	48.22 ± 2.07	0.03 ± 0.00	
SEM	2.19	0.03	
***Total rumen microorganisms***			
Buffalo	84.14 ± 2.90^a^	0.10 ± 0.00^a^	
Cow	75.29 ± 3.29^b^	0.02 ± 0.00^b^	
SEM	3.20	0.00	

a,b Values within each column with different letters are significantly different (*p* < 0.05).

**Table 3:** Protozoa genera in the rumen of water buffalo and Holstein cow (appearance percentage). Data are presented as mean

**Protozoa**	**Dry matter (%)**	**Neutral detergent fiber (%)**
***Total protozoa number***	3.68 × 105^a^	2.18 × 105^b^
*** Percentage of each protozoa***		
***Diplodinium***	41.27 b	35.70^c^
***Entodinium***	42.35^b^	48.77^a^
***Epidinium***	5.31^d^	0.00^h^
***Ophryoscolex***	0.68^g^	3.75^e^
***Eudiplodinium***	1.45^f^	1.00^g^
***Polyplastron***	1.15^f^	0.6 1^g^
***Diploplastron***	0.62^g^	0.2 5^h^
***Dasytricha***	3.47^e^	3.04^e^
***Isotricha***	3.70^e^	6.88^d^
**SEM**	0.72	0.72

a-h Values within each column with different letters are significantly different (*p* < 0.05).

The result showed that *Diplodiniinae* in buffalo rumen was more than that of cow under the same diet. *Epidinium *genera (*E. cuadatum and E. ecuadatom*) and *Diplodinium crystagali *did not exist in rumen of cattle but observed in buffalo rumen. Also, the result showed that *Ophryoscolex purkini was *found in buffalo rumen, however, in cow was more than buffalo*. *Buffalo possessed significantly higher concentrations of *Epidinium* spp., *E. maggii*, *E. bursa*, and *Diplodinium cristagalli* spp. The *E. cuadatum* and *E. ecuadatum* was absent in cow.

## Discussion

The results showed that there was no significance for *in vitro* DM, and significance for NDF and ADF digestibility between rumen protozoa of buffalo and cow. This results was in agreement with Hungate *et al.* who reported more and faster rates of fermentation for buffalo rumen microflora than in cattle.^22^ Hussain and Cheeke offered annual ryegrass straw and maize juice silage and found digestibility of NDF to be significantly higher in water buffalo than in Hereford cattle.^23^ Protozoa ciliates are capable of degrading structural polysaccharides by the ingestion and digestion with their own enzymes or by engulfment of cellulolytic bacteria retained in the vacuoles organelles, probably lysosomes.^24^^,^^25^ Preliminary investigations, however, indicated that ruminal protozoal populations differed both quantitatively and qualitatively between bison and cattle. Gupta *et al.* verified higher *in vitro* cellulose digestion in the rumen fluid of buffalo faunated (content protozoa) than that in ciliate free.^15^^,^^26^ Jouany and Senaud observed that there was a significant increase in the digestibility of lignocellulose (3 to 10%) due to the presence of rumen ciliate protozoa, particularly *Polyplastron multivesiculatum*.^1^ Demeyer reported that it is possible that in the absence of rumen protozoa a larger proportion of fiber is digested in the large intestine and caecum.^27^ The protozoa would be responsible for 34.00% of total rumen microbial fiber digestion. Different rumen protozoa concentrations have been observed between buffalo and cattle at different regions on many feeding system and in the same environment and feeding. Gupta *et al.* and, Ichhponani and Sidhu observed considerably higher *in vitro *digestibility of cellulose in buffalo than in cattle.^26^^,^^28^ These results appear contradictory, but some of this variability may be due to the different microbiological procedures and experimental conditions between groups. Kennedy observed a faster degradation rate of cellulose in the rumen of buffalo than cattle, but whole tract digestibility was not affected by animal species because fractional outflow rate was also faster in buffalo.^2^ Differences in the rumen environment among animal species may affect the microbial population and/or the activity of degrading enzymes. 

However, other researchers reported that, cattle showed higher digestibility of NDF than buffalo (0.54 *v. *0.51, *p* < 0.05). Similarly, Kennedy *et al.* in a comparative study between the Swamp buffalo and crossbreed *Bos indicus *and *Bos taurus *cattle, offered a fibrous diet and found that NDF digestibility was lower in buffalo.^29^ They reported the higher values for digestibility of NDF in cattle that was mainly due to a better utilization of cellulose compared to buffalo (0.62 *vs. *0.50, *p *< 0.05). Also, Norton *et al.* found a better digestibility of cellulose by Shorthorn cattle compared to swamp buffalo (0.58 *vs. *0.56) which was fed with a diet based on sorghum hay *ad libitum*.^3^

The results of the present study indicated that the volume potential of gas production by water buffalo rumen protozoa was higher than cow (*p *> 0.05). The differences in buffalo and cattle rumen fermentation can be explained with a different microbial activity of the two ruminant species, because of different amount of microbial population. It has also been reported that differences in fermentation patterns and gas production between the buffalo and cow might be due to the presence of different species and fermentative capacity of rumen micro-organisms such as protozoa, or to differences in their microbial activity.^30^^,^^31^ Wanapat *et al.* observed higher rates of *in vitro* fermentation in rumen fluid from water buffalo than from cow.^6^^,^^10^ Digestibility of fiber in the sugarcane pith was consistently higher by the buffalo rumen protozoa than cattle, as in the studies of Ichhponani, also more cellulose was digested by rumen fluid of water buffalo than cow.^32^^,^^33^ Therefore, rumen of the buffalo harbored active populations of ciliate protozoa than those of cows.^28^

The results of the present study showed that *Diplodiniinae* in buffalo rumen was more than cow under the same diet, that was in agreement with Franzolin and Dehority, Franzolin and Franzolin, and Franzolin *et al.* who observed a higher proportion of *Diplodiniinae*,^34^ compared to genus *Entodinium* in buffalo than cattle fed with the same conditions.^8^^,^^35^^,^^36^ According to Dehority, species of protozoa of the genus *Entodinium* comprise predominant (around 88.00 to 90.00%) rumen protozoa population for most domestic ruminants under different feeding systems, which was in agreement with the present study.^21^

*Epidinium *genera (*E. cuadatum and E. ecuadatom*) and *D. crystagali *did not exist in rumen of cattle, however, observed in buffalo rumen. The results of the present study showed *O. purkini was *found in buffalo rumen, but in cow was more than buffalo*. Diplodinium* and *Epidinium* were most commonly found in the rumen liquor of the water buffalo (Table 3). Buffalo possessed significantly higher concentrations of *Epidinium* spp., *E. maggii*, *E. bursa*, and *D. cristagalli* spp. The *Epidinium cuadatum* and *Epidinium ecuadatum* were absent in cow. But Bhatia *et al.* and Singh *et al.* reported that cattle bears *E. ecuadatom* but not buffalo, also cattle did not have *D. crystagali*, whereas there was not any *E. ecuadatom* and *O. purkini* in rumen of buffalo. It is approved that *Epidinium* was against *O. purkini*.^7^^,^^36^^,^^37^ The observation of *E. ecuadatom* and *O. purkini* in buffalo rumen was in contrary to Singh* et al.,* and absence of *D. crystagali* in rumen fluid of cattle was in agreement with those previously recorded by Singh* et al*.^38^ The study of Dehority in Brazil showed that *Ophryoscolex* did not exist in buffalo rumen.^21^ The buffalo had higher representation of the *Diplodinium* genus than cow, whereas *Entodinium* genus in cow was higher than buffalo. Gonzalez *et al.* reported that *diplodinia* in rumen fluid of buffalo was more than zebu cow.^34^ Large *Ophyroscolecidae* such as *Epidinium, Polyplastron*, and *Eudiplodinium* have higher levels of endoglucanase and xylanase activity.^39^

The ciliate protozoan *E. ecaudatum* belongs to the most common species of ciliates inhabiting the rumen of domestic ruminants.^35^^,^^39^ Coleman showed that the ability of *E. ecaudatum* to degrade microcrystalline cellulose was higher than activity of *E. maggii *and Williams.^14^ Coleman stated that xylanolytic activity of *E. ecaudatum* was comparable to that extracted from the cells of *P. multivasiculatum* and *E. maggii*.^14^ On the other hand, there are also evidence that the xylan and especially micro-crystalline cellulose digesting and fermenting capacities of *Epidinium* isolated from the rumen are substantially lower than *E. maggii* and *P. multivesiculatum*.^15^^,^^22^

In conclusion, *in vitro* fiber digestion activity and gas production of rumen protozoa of Khuzestan water buffalo was higher in comparison with Holstein cow and rumen protozoa of Khuzestan buffalo are further and more varied than those of cow under the same diet., Further studies are necessary to evaluate the protozoa role in fiber digestion for different species of ruminants in different diets.
